# High-resolution transcriptional profiling of *Anopheles gambiae* spermatogenesis reveals mechanisms of sex chromosome regulation

**DOI:** 10.1038/s41598-019-51181-1

**Published:** 2019-10-16

**Authors:** Chrysanthi Taxiarchi, Nace Kranjc, Antonios Kriezis, Kyros Kyrou, Federica Bernardini, Steven Russell, Tony Nolan, Andrea Crisanti, Roberto Galizi

**Affiliations:** 1 Department of Life Sciences, Imperial College London, London, UK; 2 Department of Experimental Medicine, University of Perugia, Perugia, Italy; 3 Department of Genetics, University of Cambridge, Cambridge, UK; 4 Department of Vector Biology, Liverpool School of Tropical Medicine, Liverpool, UK

**Keywords:** Genetic engineering, Gene expression

## Abstract

Although of high priority for the development of genetic tools to control malaria-transmitting mosquitoes, only a few germline-specific regulatory regions have been characterised to date and the presence of global regulatory mechanisms, such as dosage compensation and meiotic sex chromosome inactivation (MSCI), are mostly assumed from transcriptomic analyses of reproductive tissues or whole gonads. In such studies, samples include a significant portion of somatic tissues inevitably complicating the reconstruction of a defined transcriptional map of gametogenesis. By exploiting recent advances in transgenic technologies and gene editing tools, combined with fluorescence-activated cell sorting and RNA sequencing, we have separated four distinct cell lineages from the *Anopheles gambiae* male gonads: premeiotic, meiotic (primary and secondary spermatocytes) and postmeiotic. By comparing the overall expression levels of *X*-linked and autosomal genes across the four populations, we revealed a striking transcriptional repression of the *X* chromosome coincident with the meiotic phase, classifiable as MSCI, and highlighted genes that may evade silencing. In addition, chromosome-wide median expression ratios of the premeiotic population confirmed the absence of dosage compensation in the male germline. Applying differential expression analysis, we highlighted genes and transcript isoforms enriched at specific timepoints and reconstructed the expression dynamics of the main biological processes regulating the key stages of sperm development and maturation. We generated the first transcriptomic atlas of *A. gambiae* spermatogenesis that will expand the available toolbox for the genetic engineering of vector control technologies. We also describe an innovative and multidimensional approach to isolate specific cell lineages that can be used for the targeted analysis of other *A. gambiae* organs or transferred to other medically relevant species and model organisms.

## Introduction

The majority of the current and most promising methods for the genetic control of pest and vector populations, including the malaria-transmitting mosquitoes *Anopheles gambiae*, rely on the expression of nucleases at specific stages of gametogenesis in one, usually the male^1–5^, or both sexes^1,3,6–8^. Homing-based gene drives, for example, require expression of the homing endonuclease gene (HEG) within diploid premeiotic germ cells and exploit the cellular DNA repair machinery to copy their coding sequence into the homologous wild-type target chromosome via homology-directed repair (HDR)^1,6–9^. We and others have shown that leaky expression of the endonuclease may severely impact the effectiveness of these strategies by reducing the fitness of mosquitoes carrying the driving elements^10–13^. Persistent expression at late stages of spermatogenesis can also result in deposition of the endonuclease into the fertilised embryo, leading to lethality^4,14^ or production of resistant mutations at a high rate^10,12,15^. Other strategies based on meiotic drive elements act by impairing the transmission of gametes during meiosis, for example, through the selective removal of *X*-bearing sperm to generate extremely male-biased progenies^4,5,16^. In this case, the sex distorter element may be linked to the *Y* chromosome to increase its frequency in the population whilst being specifically active during meiosis when the haploid gametes are formed^8,17,18^. However, we have shown previously that transcriptional silencing mechanisms may impede the expression of transgenes from the sex chromosomes^4,19^. Although not yet demonstrated, this may be due to the epigenetic silencing of the *X* and *Y* chromosomes occurring exclusively in cells that undergo meiosis and generally denoted as meiotic sex chromosome inactivation (MSCI). Studies in other organisms revealed that MSCI is a specific case of a more general phenomenon responsible for the transcriptional silencing of unsynapsed chromosomes or their heteromorphic regions that fail to establish meiotic pairing, also defined as meiotic silencing of unsynapsed chromatin (MSUC)^20^.

Whilst MSCI and MSUC have emerged as novel paradigms for studying epigenetic regulation, the mechanisms that regulate gene expression in the germline remain elusive across the insect taxa outside the well-studied *Drosophila* model and appear to vary considerably across different species^21^. Meiotic silencing may have evolved to counter the spread of selfish genetic elements, such as natural sex ratio distorters and transposable elements, or to limit nonhomologous recombination between heteromorphic chromosomes. A distinct mechanism seems to regulate the transcription of the *X* chromosome in the male germline of *Drosophila melanogaster*, involving both chromosome-wide epigenetic transcriptional suppression (about 2–4 fold compared to the soma) and compensatory adaptation of sex-linked genes through the recruitment of regulatory elements that counteract the epigenetic silencing^22^. All previous attempts to obtain transcriptional profiles from meiotic and premeiotic germline stages of insect models have relied on microdissection of gonadal tissues^23,24^. These samples also included somatic cells that hindered the specific analysis of the enclosed germline cells, an essential prerequisite for a comprehensive characterisation of dosage compensation and MSCI^23,25–30^, or the selection of germline stage-specific genes and their regulatory sequences.

Significant progress has been made over the last decade towards the development of novel tools aiming to control *A. gambiae* populations. The flexibility offered by CRISPR-based nucleases has facilitated the development of gene editing and reverse genetics techniques. Nevertheless, the precise regulation of nuclease expression remains a cornerstone for the successful development of most of these strategies. The selection of functional germline promoters and the understanding of the mechanisms of gene regulation is currently very limited for the malaria vectors, relying on the transcriptomic analysis of entire germline tissues and/or orthology relationships with *Drosophila*. Seeking to fill this knowledge gap, we developed an innovative method combining the use of transgenic markers and fluorescence-activated cell sorting (FACS) to separate distinct populations of germline cells according to their progression throughout spermatogenesis. Using this approach, we generated the first transcriptomic atlas of *A. gambiae* spermatogenesis.

## Results and Discussion

### Differential labelling of mosquito male germline cells with meiotic and premeiotic fluorescent markers

**Figure 1:**
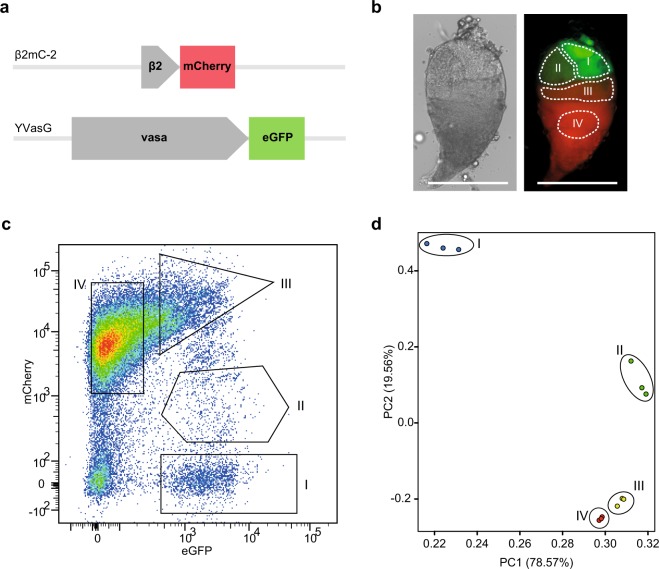
Isolation and RNA sequencing of germline cell populations from *A. gambiae* male gonads. **(a)** Schematic representation of marker transcription units utilised to identify differentiating germline cell populations. The β2:mCherry construct, containing the mCherry fluorescent marker sequence under the transcriptional control of the male meiotic β2 *tubulin* promoter, was used to generate the β2mC-2 transgenic line. The vasa2:eGFP construct, containing the eGFP sequence under the *vasa2* promoter, was previously used to generate the YVasG transgenic line^34^. The construct used to generate the β2mC transgenic lines also contains piggyBac inverted repeats and a 3xP3 promoter expressing the DsRed marker (3xP3:DsRed) for the selection of single integration events, whilst the YVasG line expresses 3xP3:eCFP (not shown here). β2mC-2 and YVasG transgenic lines were crossed to generate male individuals expressing both β2:mCherry and vasa2:eGFP in the germline. **(b)** Brightfield and overlay microscopy images showing the distribution of red and green fluorescence in β2mC-2^+^/YVasG^+^ dissected testis. Based on combined fluorescence intensity of the red and green marker from each germline cell, four areas (demarcated with dotted lines) were identified as corresponding to the premeiotic (I), meiotic (II and III) and postmeiotic (IV) germ cells. Scale bar: 200 μm. **(c)** Flow cytometry dot plot of red (mCherry) and green (eGFP) fluorescence (arbitrary units) of live germ cell suspensions obtained from β2mC-2^+^/YVasG^+^ testes, displayed using FlowJo biexponential scaling. The four populations were further analysed and separated according to the expression of the transgenic markers, cell size and granularity (Supplementary Fig. S2). **(d)** Two-dimensional Principal Component Analysis plot from RNA sequencing raw counts showing gene expression clustering of the three independent biological replicates sorted for each cell population.

Previous studies have shown how the differentiation of progenitor germ cells into mature spermatozoa progresses along the longitudinal axis of the *A. gambiae* testis^31^. Cells at different developmental stages occupy well-defined regions of the organ that can be visualised with florescent markers under the control of stage-specific promoters^32,33^. In our study we generated three transgenic lines containing independent *piggyBac*-mediated insertions (Supplementary Table S1) of a construct expressing the mCherry fluorescent marker under the *β*2 *tubulin* promoter, transcriptionally activated in concomitance with the first meiotic division and visible in the middle and basal area of dissected testis^32^. The β2mC-2 line, carrying an autosomal integration of the construct, was selected for further analysis due to higher intensity of mCherry fluorescence compared to the β2mC-1 line. We also recovered an insertion in the *X* chromosome (β2mC-X) that failed to show detectable levels of fluorescence in agreement with previous indications of *X*-linked transcriptional silencing during male meiosis^4,19^. Females from the β2mC-2 line were crossed to males from a previously constructed YVasG line expressing the eGFP fluorescent marker under the control of the *vasa2* promoter, visible in the apical region of the testis^34^, to generate males expressing both mCherry in meiotic and postmeiotic germline cells and eGFP during the initial stages of spermatogenesis, including the germline stem cells (Fig. 1a,b, Supplementary Fig. S1). Testis tissues were dissected from β2mC-2^+^/YVasG^+^ males and cell suspensions were generated by combining mechanical and enzymatic dissociation, before being subjected to fluorescence-activated cell sorting (FACS).

### Flow Cytometry purification and RNA sequencing of premeiotic, meiotic and postmeiotic germline populations

Cell viability, intensity of the fluorescent germline markers, cell size and granularity were all used as criteria to isolate four cell populations that were hypothesised to include mitotically dividing germline stem cells (GSCs) and spermatogonia in population I, meiotic primary and secondary spermatocytes in population II and III, and the postmeiotic spermatids in population IV (Fig. 1c, Supplementary Fig. S2). The FACS fractionation was performed on three independent biological replicates yielding a minimum of 2817 to a maximum of 10127 cells for each sample. These were processed for RNA sequencing to obtain between 27.8 to 51.8 million 100 bp paired-end reads after quality filtering steps. Principal Component Analysis (PCA) on raw counts confirmed the existence of clearly distinct transcriptional profiles in the sorted populations, predominantly differentiating between the premeiotic and meiotic samples. Besides showing tight concordance between the three biological replicates, we observed close transcriptional correlation between the putative late meiotic and postmeiotic groups (III and IV respectively), whereas groups I and II were transcriptionally distinct between each other and the postmeiotic populations (Fig. 1d, Supplementary Table S2).

### Analysis of meiotic sex chromosome inactivation and dosage compensation in the *Anopheles gambiae* male germline

**Figure 2:**
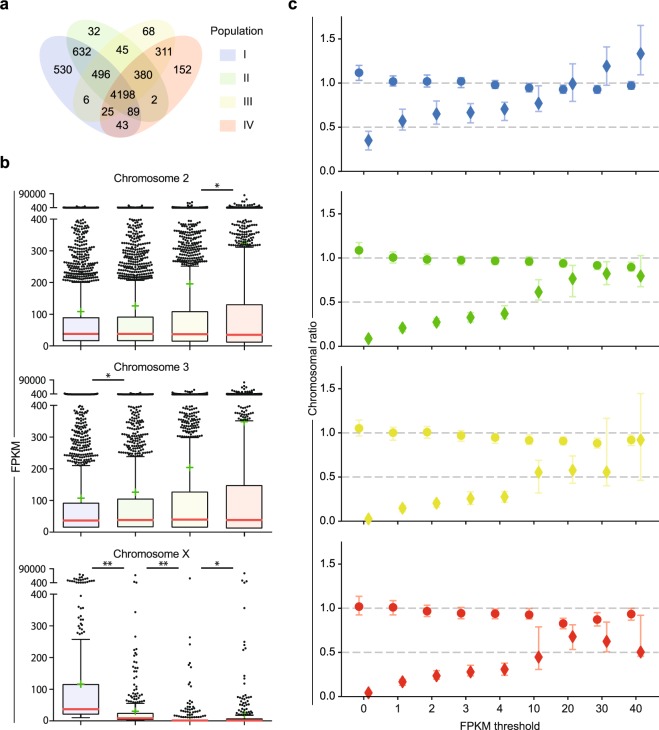
Chromosome mapping, developmental stage distribution and expression ratios of genes transcribed in testis populations. (**a**) Venn diagram showing the number of genes expressed within and across the four germline populations. (**b**) Box plots showing the transcript abundance of genes mapping on chromosome *2*, *3* and *X* for each population. Boxes extend from the 25^th^ and the 75^th^ percentile of FPKM values, with the median values represented as horizontal red lines. Error bars represent the standard deviation (SD) and mean values are indicated with green cross marks. Significance between chromosome medians from consecutive populations was calculated using the Wilcoxon rank-sum test (“*” corresponds to *P* < 0.05 and “**” to *P* < 0.0001). Only transcripts with level of expression higher than 10 FPKM in at least one population were used in “a” and “b” (**c**) Chromosome-wide median expression ratios between *X* chromosome and autosomes (rhombus) and between autosomes (circles) as a function of increasing FPKM thresholds. Vertical bars indicate 95% confidence intervals. Population I is indicated in blue, II in green, III in yellow and IV in red.

In order to reduce noise after read assembly, we introduced a minimum cut-off for each gene of 10 fragments per kilobase of transcript per million mapped reads (FPKM) resulting in a total of 7,009 annotated genes with transcript levels above threshold in at least one of the four populations. We identified 6,019 genes expressed in population I, 5,874 in II, 5,529 in III and 5,200 in IV, with 4,198 genes showing expression across all four populations (Fig. 2a, Supplementary File S1). As the cell separation was also based on the intensity of the fluorescent markers, we confirmed the positive correlation between the expression of the eGFP and mCherry transgenes and the endogenous *vasa* and *β2 tubulin* genes, transcribed under the same *cis*-regulatory elements (Supplementary Fig. S3).

We observed that only 32% of the currently annotated *X*-linked genes were expressed in at least one of the populations (350 genes from a total of 1090), compared to the 55% and 54% from chromosomes *2* and *3* respectively, confirming the underrepresentation of male-germline genes located on the *X* chromosome (Chi squared *P* < 0.0001)^19^. The analysis of the median expression of annotated genes located on each chromosome highlighted an extremely significant reduction (Wilcoxon rank-sum *P* < 0.0001) of *X*-linked genes expression in the premeiotic population I, showing a median value of 37.0 log2(FPKM), compared to the meiotic population II (8.1 log2(FPKM)) and III (1.5 log2(FPKM)), as well as the postmeiotic population IV (1.9 log2(FPKM)). However, the median expression of autosomal genes was not negatively impacted across the four germline groups, supporting the MSCI hypothesis to explain the transcriptional repression of the *X* chromosome. On the other hand, whilst the mean expression of *X*-linked genes shows a reduction after the premeiotic stages, the mean expression of autosomal genes gradually increases during sperm development confirming that the majority of the genes that are highly expressed in the testis are located in the autosomes and may have a role during the meiotic and postmeiotic stages (Fig. 2b).

Besides ruling out the presence of wide *X* inactivation throughout male gametogenesis in favour of MSCI mechanisms, we were able to analyse the presence of dosage compensation across spermatogenesis and its impact within each developmental stage. We used the median expression value of each chromosome to calculate the ratio between *X* chromosome and autosomes (*X:A*) and between the two autosomes (*2:3*) applying a range of FPKM thresholds from zero to 40. Considering that males have only one *X* chromosome compared to two copies of each autosome, if compensation does not occur, we would expect a *X:A* ratio of 0.5 (assuming a *2:3* ratio equal to 1) in the premeiotic population I where the *X* chromosome expression is not affected by MSCI. Absence of dosage compensation and presence of MSCI in the meiotic and postmeiotic groups would instead result in *X:A* ratios below 0.5. Whilst chromosome *2:3* expression ratio oscillates around 1 across all the germline stages, the *X:A* ratio in the premeiotic population I ranges from a minimum of 0.35, when all the expressed genes are included (FPKM > 0), to 0.71 when a minimum threshold of 4 FPKM is applied, indicating the absence of both dosage compensation and transcriptional repression of the *X* chromosome in the premeiotic germline stage. The inclusion of genes with very low expression (below 1 FPKM) is expected to bias the analysis of dosage compensation, lowering the *X:A* ratio, as most *X*-linked genes are scarcely expressed in the germline^19^. In contrast, a low number of *X*-linked genes, highly transcribed in the premeiotic population (FPKM > 10), show average expression close to or higher than the autosomal counterpart, possibly indicating the presence of compensation (or overcompensation for FPKM > 30) mechanisms^35,36^ that may be acting to counterbalance MSCI and maintain transcripts available during the transcriptionally silenced meiotic and postmeiotic stages. Furthermore, the effect of meiotic silencing is reflected on the meiotic and postmeiotic *X:A* ratios reaching values close to zero, when low thresholds are applied, and greater than 0.5 only when thresholds above 10 FPKM are applied (Fig. 2c, Supplementary Table S3).

### Differential expression analysis of germline stage-enriched genes and novel transcripts

**Figure 3:**
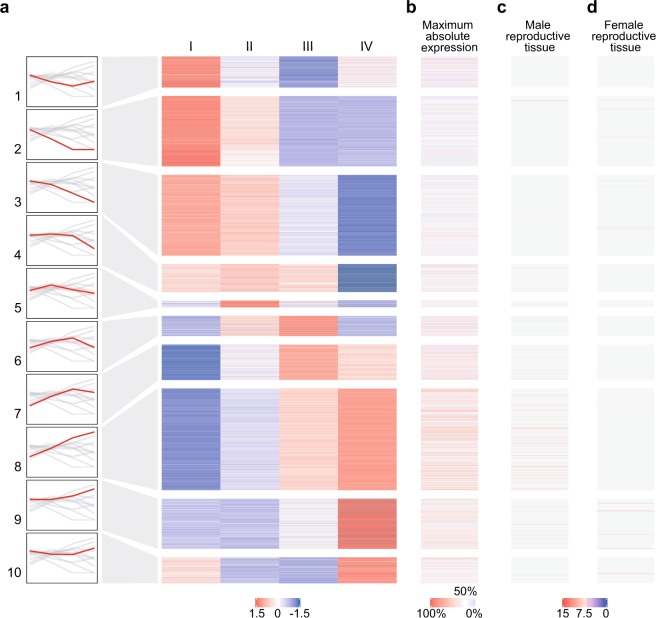
Transcriptional profiling of annotated genes that are differentially expressed across the four germline populations. (**a**) False-colour k-means clustering heatmap summarizing the expression patterns (as Z-score of log2(FPKM + 1)) of annotated genes that are differentially expressed (*P* < 0.05 according to LRT) and have expression levels above 10 FPKM in at least one of the populations. The diagrams on the left report the expression trend of annotated genes for each cluster as log2(FPKM + 1). **(b)** Maximum absolute expression of each gene based on the highest log2(FPKM + 1) value detected across the four populations. **(c)** Absolute expression (as arbitrary unit of fluorescence intensity) of germline-enriched genes in adult males and **(d)** germline-enriched genes in adult females defined using MozAtlas microarray data^27^ (tau-value ≥ 0.8). Z-score and expression values are displayed according to colour codes to indicate: high levels (red) and low levels (blue) of expression, the 50^th^ percentile (white), non-testis-enriched or non-ovary-enriched genes based on MozAtlas τ < 0.8 (grey) and not-applicable data from MozAtlas (light yellow).

Annotated genes with expression higher than 10 FPKM in at least one of the four populations were analysed for differential expression across the developmental stages by applying a likelihood ratio test (LRT). A total of 6872 genes were identified as differentially expressed and K-means clustering based on Z-Score ranking was used to define ten different clusters, grouped according to similarity of their expression profile. Among these, 1740 genes show germline enrichment based on the *Baker et al*. gene expression dataset^37^ with 1139 genes being testis-enriched, 354 ovary-enriched and 247 germline-enriched for both sexes. Interestingly, many of the testis-enriched genes are highly transcribed in the late meiotic and postmeiotic clusters, suggesting that the formation of distinctive sperm structures relies on the cooperation of a number of testis-specific genes (Fig. 3, Supplementary File S1). A similar pattern of expression was recently described in other organisms, including *D. melanogaster*^24,38^, and it is also consistent with the increasing mean expression of genes located in chromosome *2* and *3* (Fig. 2b). We further investigated the expression pattern of meiotic genes that are highly conserved among eukaryotic taxa and have annotated orthologues in *A. gambiae*^39^. *Msh4* (AGAP012245), *Msh5* (AGAP002642) and *Spo11* (AGAP010898), are indeed included in the clusters 4, 6 and 7 with peak of expression in the meiotic stages. Orthologues of *Drosophila* testis genes, previously analysed via *in situ* hybridisation^24^, also show similar expression patterns in our dataset. Specifically, AGAP010253 (orthologue of *Prosalpha6*) and AGAP000261 (CG7556) in cluster 2, AGAP010672 (*SdhC*) and AGAP013212 (CG1409) in cluster 9, AGAP001744 (CG3803) in cluster 3 and AGAP005421 (CG6404) which does not show a significant differential expression as for its *Drosophila* orthologue (Supplementary Fig. S4).

**Figure 4:**
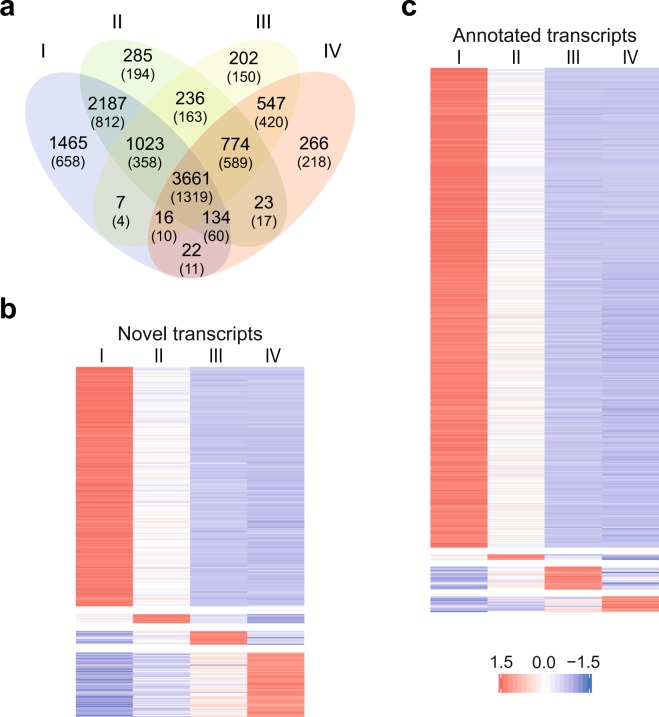
*De novo* transcriptome profiling of germline populations. (**a**) Venn diagram showing the number of transcripts identified via *de novo* transcriptome analysis of the four populations. The number of novel transcripts (not yet annotated in the AgamP4.8 genome) is given between parentheses. **(b)** False-colour k-means clustering heatmap showing the expression as Z-score of log2(FPKM + 1) of non-annotated (novel) transcripts and **(c)** annotated transcripts that are enriched in each of the four populations (fold change > 2 compared to each of the other three populations and adjusted *P*-value < 0.05). Only genes that showed expression above 1 FPKM in at least one of the cell populations were included in the *de novo* transcriptome analysis.

The RNA-seq dataset was further interrogated to identify unannotated genes that may be differentially expressed as well as alternative transcripts that may be differentially used during spermatogenesis. We therefore performed *de novo* transcriptome analysis and identified 4,983 novel transcripts not yet annotated in the AgamP4.8 genome. After applying differential expression analysis, we found a total of 925 differentially enriched transcripts, 238 of these were identified as novel transcript isoforms mapping within previously annotated genes whilst 127 aligned to non-annotated regions of the AgamP4.8 genome (Fig. 4, Supplementary File S2). Between these we were able to pinpoint significantly switched transcript isoforms, such as alternative splicing or alternative transcription start or termination site, that are differentially used at specific timepoint of the male gametogenesis (Supplementary Fig. S5).

**Figure 5:**
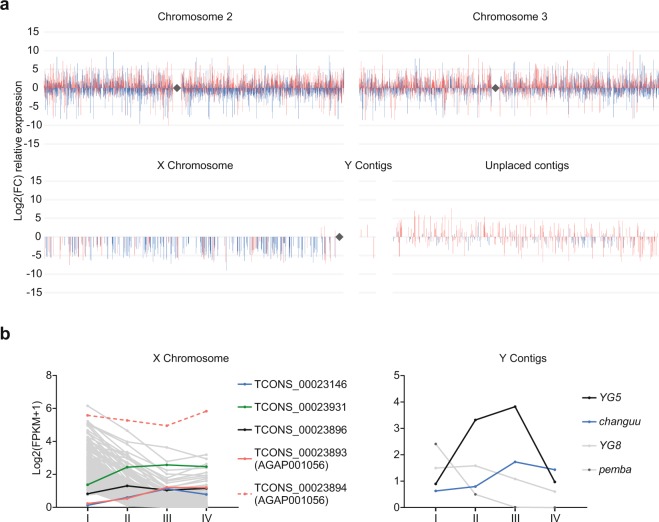
Differentially expressed transcripts between meiotic and premeiotic stages. (**a**) Spatial mapping of transcripts relative expression, shown as log2 fold change, between the meiotic population III and the premeiotic population I across the *A. gambiae* genome. Transcripts not yet annotated in the current version of the genome (AgamP4.8) are indicated in red and annotated transcripts are in blue. Transcripts are ordered based on their start position on each chromosome whereas gaps between bars indicate transcripts below 1 FPKM or not differentially expressed (adjusted *P* > 0.05). Rhombus symbols indicate the centromeres. **(b)** Expression profiles as log2(FPKM + 1) of meiosis-enriched transcripts (coloured lines) located on the *X* chromosome (left) and *Y* contigs (right) showing higher expression in the meiotic population III compared to the premeiotic population I and therefore positive fold change in Fig. 5a. Dashed lines indicate the alternative transcript isoform and grey lines indicate transcripts with negative fold change. Only transcripts above 1 FPKM in at least one of the populations are shown.

Though a reduced expression of *X*-linked genes in the meiotic clusters was apparent, we further analysed the meiotic enrichment of annotated and non-annotated transcripts across the *A. gambiae* genome. We found a homogeneous distribution in chromosome *2* and *3*, whilst the *X* chromosome confirmed a paucity of meiotic genes. Nonetheless, four transcripts located in proximity to the centromeric region of the *X* chromosome show a positive meiotic/premeiotic fold change, suggesting a meiotic role and possible evasion of MSCI at this specific genomic region (Fig. 5). One of these genes, previously annotated as AGAP001056, is a highly conserved ubiquitin-conjugating enzyme that may have a role in epigenetic regulation of the sex chromosomes^20,40,41^. Similarly, two *Y*-linked transcripts, previously described as associated and in multiple copies (*YG5* and *changuu*)^42^, show an enrichment of expression in the meiotic stage of 4 and 11 fold respectively, whilst the other two *Y* genes expressed above threshold (*YG8* and *pemba*) show decreasing levels of transcripts (Fig. 5b). Previous studies highlighted a high degree of similarity between *Y*-associated and *X*-associated repetitive sequences positioned at the proximal end of the *X* chromosome indicating possible meiotic pairing and crossing-over between the sex chromosomes at these regions^42^. The proximal end of the *X* chromosome also contains the ribosomal DNA (rDNA) locus, which defines the molecular forms of *A. gambiae* species^43^. In *Drosophila* male meiosis, segregation of the *X* and *Y* chromosomes relies upon pairing between the proximal *X* heterochromatin and the short arm of the *Y* chromosome, respectively located nearby the *X* and *Y* rDNA arrays. Transgenic studies have also demonstrated that this pairing site consists mainly of repeats within the rDNA intergenic sequences which also contain RNA polymerase I promoters. One hypothesis is that these regulatory sequences must remain transcriptionally active to allow chromosomal pairing, therefore suggesting a possible correlation between pairing and transcription^44–46^.

### Biological process and metabolic pathway analysis

We performed gene ontology (GO) term enrichment analysis to highlight the biological role of genes expressed at different timepoints of *A. gambiae* spermatogenesis. We used the PANTHER online tool to interrogate the gene groups, previously clustered according to differential expression profiles and summarised in Fig. 3, as well as all the testes-enriched genes irrespectively of their temporal expression across spermatogenesis.

GO analysis of differentially expressed genes highlighted a significant enrichment for RNA processing, cytoskeleton organisation, ribosome biogenesis and translation-related terms distinctive of differentiating GSCs, for the clusters showing predominant premeiotic expression (1, 2 and 3). On the other hand, genes up-regulated in the postmeiotic population, grouped in clusters 9 and 10, show a significant overrepresentation of metabolic enzymes and mitochondrial activity related terms typical of postmeiotic sperm maturation where energy production, in the form of adenosine triphosphate (ATP), is provided via oxidative phosphorylation. Interestingly, the most overrepresented GO in the meiotic and postmeiotic cluster 8 is associated with microtubule-based movement, which is essential for sperm motility (Supplementary Fig. S6, Supplementary File S3).

Unfiltered GO analysis of all the testes-enriched genes highlighted a peculiar overrepresentation of microtubule-associated terms whose expression profiles confirmed their requirement in the late-meiotic and postmeiotic stages. Other terms highly overrepresented are related to protein modification and ubiquitination functions, including genes that are predominantly transcribed in the premeiotic and postmeiotic stages, DNA repair and cellular response to DNA damage, which are mostly required in the initial stages of spermatogenesis as confirmed by their trends of expression in our dataset (Supplementary Fig. S7, Supplementary File S3). These results indicate that highly specialised genes, most of which are testis-specific or testis-enriched, are required to accomplish these crucial biological processes during sperm formation.

We also reconstructed the expression dynamic of the key KEGG metabolic pathways involved in male gametogenesis^47^. Genes included within the ubiquitin-mediated proteolysis pathway show a characteristic trend of expression in the premeiotic stage, where ubiquitination is required for GSCs maintenance^48–51^, and high transcript levels in the postmeiotic stage, where these components are necessary for sperm individualisation, reorganisation of chromatin structure and nuclear condensation, acrosome formation and paternal mitochondrial elimination^24,52^. On the other hand, oxidative phosphorylation and citrate cycle components, predominantly transcribed in the postmeiotic population, utilise the products of both nuclear and mitochondrial genes to provide the energy required in the late stages of spermatogenesis and mature sperm^53,54^. We finally show that most of the genes involved in DNA replication, RNA transcription (within the Basal transcription factors and RNA polymerase pathways), RNA maturation (Spliceosome) and translation (Ribosome) are already present in the initial stages of spermatogenesis (Supplementary Fig. S8) confirming that most of the genes required for the late stages are transcribed before meiosis, as previously observed also in other model organisms and human spermatogenesis^24,55,56^.

## Conclusion

The rapid development of gene editing tools aimed at controlling human malaria vectors and their recent proof-of-principle demonstrations have highlighted the need for furhter studies of the mosquito gametogenesis for at least two reasons. First, numerous genetic control methods intend to interfere with mosquito reproduction, for example by targeting genes that have a role in male or female fertility. Second, the majority of the genetic control technologies currently under development require specific spatiotemporal expression of nucleases in the germline. In this work we describe a multidimensional method that combines next-generation sequencing with genetic labelling and cell-sorting technologies to recapitulate the transcriptional repertoire of *A. gambiae* spermatogenesis. This approach allowed us to separate germline cells from somatic tissues, removing potentially confounding effects and facilitating a more detailed analysis of germ cell gene expression. In addition, our fluorescence-based fractionation allowed us to unravel the temporal gene expression programme from premeiotic, through meiotic and then postmeiotic germline lineages to clearly identify some of the mechanisms orchestrating transcriptional regulation during spermatogenesis. We show the first direct evidence of meiotic chromosome silencing that specifically downregulates gene expression on the *X* chromosome from the onset of the meiotic stages. Differential expression analysis between the meiotic and premeiotic stages also highlights possible presence of MSCI escapees. We show that only very few *X* and *Y*-linked genes show a positive fold change between the meiotic population III and the premeiotic population I, with all four *X*-linked transcripts being located at the proximal end of the *X* chromosome. Further studies will be required to clarify possible correlations between chromosomal pairing and transcription at these specific loci. On the other hand, our data also confirm unequivocally that dosage compensation does not act on the *X* chromosome in diploid germline cells as indicated by previous analysis of whole testes and in contrast to somatic tissues where dosage compensation is present^27–29^.

Additionally, we provide an extensive dataset of differentially expressed genes and differentially switched transcript isoforms, some enriched at specific stages of sperm development, which represents an invaluable resource for further functional analysis. Furthermore, we described the biological role of genes expressed at specific timepoints of spermatogenesis and reconstructed the spatiotemporal expression dynamics of the main metabolic pathways involved. A deeper analysis of these data will further our understanding of the complexity of cellular differentiation during gametogenesis and facilitate the identification of the genetic components regulating male fertility in *A. gambiae*. Some of these may offer novel targets for malaria vector control applications that aim to interfere with male fertility and provide new regulatory components for the fine-tuned expression of nucleases in the mosquito male germline. We believe that the approach we developed could be applied to the study of gene expression and epigenetic mechanisms in other relevant tissues or cell lineages, such as the female germline, olfactory organs or midgut, to better understand the processes of reproduction or the molecular interactions between the parasites and their insect vectors, in *Anopheles* as well as other vector species.

In this study we describe a novel multidimensional approach that combines genetic engineering, fluorescence-activated cell sorting and RNA sequencing technologies to reconstruct for the first time the transcriptional atlas of the *Anopheles gambiae* male germline. Besides providing a detailed transcript composition and biological function of genes expressed in the key stages of spermatogenesis, we were able to: elucidate the as yet mysterious mechanisms regulating *A. gambiae* spermatogenesis, provide a novel toolbox applicable to the study of other mosquito organs or other organisms and generate a highly valuable dataset that can offer novel targets as well as regulatory components for the genetic control of malaria-transmitting mosquitoes.

## Methods

### Generation of the β2mC transformation construct

The coding region of mCherry was amplified using primers containing XhoI (TTTCTCGAGATGGTGAGCAAGGGCGAG) and PacI (CCTTAATTAATTACTTGTACAGCTCGTCCATGC) from a plasmid generated previously in our laboratory and was subcloned into the attB-eCFP(β2-β3’UTR) vector containing the regulatory regions of the *β*2 *tubulin* gene. The β2:mCherry expression cassette was then subcloned by using FseI into the pBac(3xP3:DsRed) vector containing the 3xP3:DsRed cassette flanked by *piggyBac* inverted repeats to obtain the final germline transformation construct pBac(3xP3:DsRed)(β2:mCherry) that was used to generate the β2mC transgenic lines.

### Generation and characterisation of transgenic mosquito lines

A Femtojet Express injector and a Narishige 202ND micromanipulator mounted on a Nikon TE-DH100W inverted microscope were used to inject ~300* A. gambiae sensu stricto* embryos (here referred as wild-type) with a mixture of 0.2 μg μl−1 of the pBac(3xP3:DsRed)(β2:mCherry) plasmid and 0.4 μg μl−1 of a *vasa*-driven piggyBac transposase helper plasmid. Transient expression of the DsRed marker was analysed from the hatched larvae with a Nikon inverted microscope (Eclipse TE200) at a wavelength of 563 nm (Filter 630/30 nm emission, 595 nm dichroic). DsRed positive mosquitoes were individually crossed to wild-type and a total of three transgenic lines originated from single integration events (named as β2mC-1, β2mC-2 and β2mC-X) were selected by inverse PCR and ~50% mendelian inheritance of the DsRed marker as previously described^4^. β2mC-2^+^/YVasG^+^ mosquitoes were obtained by crossing 50 YVasG virgin males^34^ and 50 virgin females of the β2mC-2 line. Fluorescence was analysed from dissected testes on an EVOS FL Imaging System (Advanced Microscopy Group) equipped with a GFP LED Light Cube (470 nm excitation and 525 nm emission) and a RFP LED Light Cube (530 nm excitation and 593 nm emission).

### Testis microdissection and cell dissociation

An average of 250 testes were dissected from 1-day old adult mosquitoes and mechanically homogenised (0.152–0.254 mm borosilicate glass homogeniser, Fisherbrand) in 50 μL DPBS (Thermo Fisher Scientific) before being transferred into an Eppendorf tube using a Hamilton syringe (SGE Analytical Science). The volume of the suspension was adjusted to 145 μl by adding DPBS and the enzymatic cell dissociation medium was assembled by adding 70 μl of trypsin 2.5% and 35 μl of collagenase 2.5%. Each sample was incubated for 15 min at room temperature on gentle shaking and then filtered through a 41 μm nylon mesh filters (Millipore) into 500 μL of Schneider’s insect medium supplemented with 10% FBS (Sigma-Aldrich). Cells were harvested via centrifugation at 425 g for 5 min at 4 °C and resuspended in Schneider’s insect medium prior to sorting. The dissection and cell dissociation procedures were repeated to obtain three independent biological replicates.

### Flow cytometry isolation of germline cell populations

After adding 50 μl per ml of TO-PRO-3 Iodide (Invitrogen), for the exclusion of dead cells (less than 10% of total events), the cell suspensions were analysed and sorted using a BD FACSAria III sorter (BD Biosciences, Flow Cytometry Facility at Imperial College London) with a 100 μm nozzle and 28 PSI pressure. The following laser and filter combinations were used for the excitation and detection: 561 nm and 610/20 nm for mCherry, 488 nm and 530/30 nm for eGFP, 488 nm and 488/10 nm for side and forward scatter area (SSC-A and FSC-A), 640 nm and 660/20 nm for TO-PRO-3 stain. The FlowJo software package (LLC) was used for the gating of the four germline cell populations based on mCherry and eGFP fluorescence intensity. Each population was subsequently gated according to SSC-A and FSC-A for cell size, granularity and internal complexity. The cells positive only for the eGFP marker and above 65 K FSC-A threshold were selected and sorted as the premeiotic population I. Cells positive for both markers were gated in two distinct populations based on the low intensity of the mCherry fluorescence and FSC-A threshold above 105 K (population II), or high mCherry intensity and above 120 K FSC-A (population III). Cells positive only for mCherry fluorescence and FSC-A values between 40 and 60 K were gated as the postmeiotic population IV. Quality and homogeneity of the four populations obtained after sorting was assessed on an inverted microscope (EVOS FL Cell Imaging System) before proceeding with the RNA extraction.

### RNA extraction, preparation of cDNA libraries and sequencing

Total RNA was extracted from the three replicates of each cell population sorted, composed of up to ~10,000 cells each, by using the PicoPure RNA Isolation Kit (Applied Biosystems) with additional DNase treatment using the RNase-Free DNase Set (Qiagen). Quality and quantity of the extracted RNA were assessed using Bioanalyzer 2100 (Agilent Technologies) before cDNA library preparation by using the SMART-Seq v4 Ultra Low Input RNA Kit (Clonetech) and the Nextera XT DNA Library Preparation Kit (Illumina). After quantification and quality inspection of the double-stranded cDNA obtained (BioAnalyzer 2100), the samples were diluted and pooled to generate two multiplexed cDNA libraries (between 3.5 and 3.6 nM). Library preparation and analysis was performed at Polo GGB (Italy). Sequencing was performed with the HiSeq 2500 System (Illumina) with paired end reads of 100 nucleotides (Genomics Facility, MRC London Institute of Medical Sciences - UK).

### Differential gene expression analysis and k-means clustering

Sequencing reads were trimmed and cropped (Trimmomatic^57^) before mapping (HISAT2^58^) to the reference genome AgamP4.8 with the addition of β2:mCherry and vasa2:eGFP plasmid sequences and *Y* contigs^42^. Uniquely mapped reads were quantified using featureCounts^59^ before being imported into DESeq2 for differential expression analysis between the four populations^60^. Likelihood ratio test was applied to determine differentially expressed genes (adjusted *P*-value below 0.05). Genes with expression above 10 FPKM in at least one population were selected and log2-transformed FPKM values were used for Z-score clustering using k-means method.

### MSCI and dosage compensation analysis

DESeq2 FPKM values of genes with FPKM above 10 in at least one population were used to calculate median, mean, percentiles and standard deviation of chromosome *2*, *3* and *X* gene expression. Wilcoxon rank-sum test was used to calculate significance between chromosome medians from consecutive populations (Fig. 2b). For the dosage compensation analysis, the “boot” package in R^61,62^ was used to calculate the *X:A* and *2:3* chromosome-wide median expression ratios and the 95% confidence intervals for each cell population. Median FPKM values of genes located in the *X* chromosome or chromosome *2* (10,000 replicates of randomly selected gene subsets) were respectively divided by the median of all autosomal genes (for *X:A* ratios) and the median of all the genes in chromosome *3* (for *2:3* ratios) applying expression thresholds of 0, 1, 2, 3, 4, 10, 20, 30 or 40 FPKM^63^ (Fig. 2c).

### Tissue-enrichment calculation

Male or female germline-enriched genes were defined by calculating the tau-value (τ)^64^ from the MozAtlas microarray dataset^37^.

$${\rm{\tau }}=\frac{{\sum }_{i=1}^{N}\,(1-{x}_{i})}{N-1};\,{x}_{i}=1-\frac{{x}_{i}}{{\rm{\max }}(x)}$$

*N* represents the number of tissues and *X*_*i*_ is the expression profile component normalised by the maximal component value. Genes with a τ close to 0 are broadly expressed across all tissues, whilst genes with tissue-enriched expression have τ close to 1. Genes showing values of τ ≥ 0.8 were selected as germline-enriched in *A. gambiae* adult males when showing maximum expression in testis, or females when showing maximum expression in ovaries samples.

### *De novo* transcriptome analysis and alternative usage of transcript isoforms

Aligned reads from HISAT2 were assembled into potential transcripts with Stringtie^65^ applying a threshold of 10 reads for coverage of exons and 15 for junctions and merged using the Cuffmerge tool (Cufflinks suite^66^). A threshold of 5 FPKM in at least one population was used for the quantification of the resulting set of transcripts using Stringtie to reduce false positives. A new gene set was created by using the gffcompare program to include novel gene transcripts to the previously annotated in the AgamP4.8 gene set. After transcript quantification with Kallisto^67^, differentially expressed isoforms were calculated using Sleuth (Pachter Lab)^68^. Enriched transcripts in each population were selected based on fold change higher than 2 compared to each of the other three populations and adjusted *P*-value for multiple testing below 0.05. A threshold of 1 FPKM in at least one population was applied to filter out transcripts with very low expression. The reduced threshold applied for the *de novo* transcriptome analysis is due to the higher number of potential transcripts present in the novel gene set resulting in the reduction of FPKM values. The R package IsoformSwitchAnalyzeR^69^ was used to identify, annotate and visualise differentially used isoforms (adjusted *P* < 0.05) among the germline populations (also called isoform switching).

### Gene ontology analysis

The PANTHER (protein annotation through evolutionary relationship) classification system^70^ was used to identify *A. gambiae* biological processes enriched in each of the germline clusters and in the group of differentially expressed genes that show enrichment in testes. Significance of the statistical overrepresentation test was analysed by applying Fisher’s Exact test with False Discovery Rate correction.

## Supplementary information

Supplementary information

Supplementary File S1, S2, S3

## Data Availability

RNA-Seq datasets will be deposited in the NCBI and VectorBase databases.
