# Supplementary material for: High-resolution transcriptional profiling of Anopheles gambiae spermatogenesis reveals mechanisms of sex chromosome regulation
Source: Sci Rep. 2019 Oct 16;9:14841. doi: 10.1038/s41598-019-51181-1 (PMC6795909; doi:10.1038/s41598-019-51181-1)
Supplement: Supplementary file 1 — Supplementary information [file 41598_2019_51181_MOESM1_ESM.pdf]

Title

High resolution transcriptional profiling of *Anopheles gambiae* spermatogenesis reveals mechanism of sex chromosome regulation

Authors

Chrysanthi Taxiarchi[12], Nace Kranjc[1], Antonios Kriezis[1], Kyros Kyrou[1], Federica Bernardini[1], Steven Russell[3], Tony Nolan[14], Andrea Crisanti[1], Roberto Galizi[1]*

Supplementary information

Supplementary Table S1: Characterisation of β2mC transgenic lines.

Transgenic line	% transgenic[1]	Insertion site[2]	Chromosome band
β2mC-1	54.4 (90)	CTTCGGATTTTTAA-PB-TTAAGCGCAAGGTT	UNKN
β2mC-2	46.6 (60)	GAGGATGAAGTTAA-PB-TTAAGTACAGTGCG	UNKN
β2mC-X	53.3 (60)	TAAAAAACCGTTAA-PB-TTAAATAAAACCAA	X-5D

(1) Percentage of transgenic offspring scored in the progeny of the first transgenic founders. The total number of larvae screened is given in parenthesis. (2) 14 base pairs flanking the piggyBac integration site and predicted chromosomal insertion based on current genome assembly.

Supplementary Table S2: Pearson correlation coefficient of all the biological replicates.

	**I Rep 1**	**I Rep 2**	**I Rep 3**	**II Rep 1**	**II Rep 2**	**II Rep 3**	**III Rep 1**	**III Rep 2**	**III Rep 3**	**IV Rep 1**	**IV Rep 2**	**IV Rep 3**
**I Rep 1**	1.0000											
**I Rep 2**	**0.9823**	1.0000										
**I Rep 3**	**0.9789**	**0.9548**	1.0000									
**II Rep 1**	0.8488	0.8294	0.7979	1.0000								
**II Rep 2**	0.7701	0.7447	0.7149	**0.9862**	1.0000							
**II Rep 3**	0.7830	0.7468	0.7419	**0.9825**	**0.9920**	1.0000						
**III Rep 1**	0.4599	0.4416	0.4000	0.8305	0.8931	0.8749	1.0000					
**III Rep 2**	0.4327	0.4147	0.3780	0.8138	0.8800	0.8621	**0.9970**	1.0000				
**III Rep 3**	0.4570	0.4359	0.4061	0.8303	0.8930	0.8835	**0.9925**	**0.9950**	1.0000			
**IV Rep 1**	0.3913	0.3669	0.3520	0.7732	0.8381	0.8263	0.9730	0.9816	0.9800	1.0000		
**IV Rep 2**	0.3783	0.3570	0.3354	0.7674	0.8343	0.8182	0.9753	0.9843	0.9791	**0.9983**	1.0000	
**IV Rep 3**	0.3848	0.3626	0.3432	0.7704	0.8376	0.8243	0.9749	0.9839	0.9818	**0.9988**	**0.9986**	1.0000

Correlation values between replicates (Rep) of the same sample are indicated in bold.

Supplementary Table S3: Chromosome-wide median expression ratios between *X* chromosome and autosomes (*X*:*A*) and between autosomes (*2*:*3*) as a function of increasing FPKM thresholds.

	Population I		Population II		Population III		Population IV	
**FPKM threshold**	***X:A***	***2:3***	***X:A***	***2:3***	***X:A***	***2:3***	***X:A***	***2:3***
**0**	0.350376 (917/10690)	1.117451 (6183/4507)	0.085872 (831/10633)	1.086674 (6118/4515)	0.025497 (686/10413)	1.051632 (5990/4423)	0.042358 (715/10057)	1.01867 (5785/4272)
**1**	0.571388 (576/7587)	1.017861 (4416/3171)	0.20808 (428/7787)	1.005417 (4506/3281)	0.146643 (209/7836)	1.002019 (4525/3311)	0.166951 (255/7531)	1.010136 (4346/3185)
**2**	0.650212 (518/7142)	1.018933 (4169/2973)	0.272494 (333/7312)	0.983543 (4246/3066)	0.203202 (152/7324)	1.008115 (4229/3095)	0.234816 (183/6920)	0.966899 (4012/2908)
**3**	0.666549 (487/6816)	1.019181 (4000/2816)	0.326781 (283/6979)	0.974556 (4071/2908)	0.256033 (114/6911)	0.969458 (3992/2919)	0.278578 (148/6512)	0.943684 (3772/2740)
**4**	0.705947 (461/6598)	0.979023 (3872/2726)	0.36937 (249/6720)	0.967305 (3924/2796)	0.27543 (101/6600)	0.947079 (3829/2771)	0.307851 (130/6167)	0.93863 (3574/2593)
**10**	0.771018 (350/5565)	0.945489 (3262/2303)	0.614083 (149/5624)	0.960344 (3277/2347)	0.553159 (51/5387)	0.915979 (3121/2266)	0.446783 (75/5029)	0.926351 (2898/2131)
**20**	0.992577 (263/4387)	0.928266 (2569/1818)	0.765333 (99/4506)	0.938786 (2612/1894)	0.576514 (31/4329)	0.908194 (2492/1837)	0.678284 (44/4108)	0.826995 (2384/1724)
**30**	1.191703 (207/3663)	0.926871 (2145/1518)	0.822087 (71/3747)	0.916534 (2167/1580)	0.555921 (24/3638)	0.882635 (2101/1537)	0.62428 (34/3541)	0.871947 (2050/1491)
**40**	1.33226 (167/3129)	0.970425 (1814/1315)	0.795713 (56/3169)	0.896114 (1830/1339)	0.920404 (16/3165)	0.919766 (1797/1368)	0.503921 (30/3108)	0.933909 (1780/1328)

Numbers in brackets indicate the fraction of genes (above the applied FPKM threshold) of the total number of annotated genes for each chromosome used to calculate ratios

Supplementary Figure S1: Fluorescent microscopy of β2mC^{+}, YVasG^{+} and β2mC-2^{+}/YVasG^{+} transgenic testes.

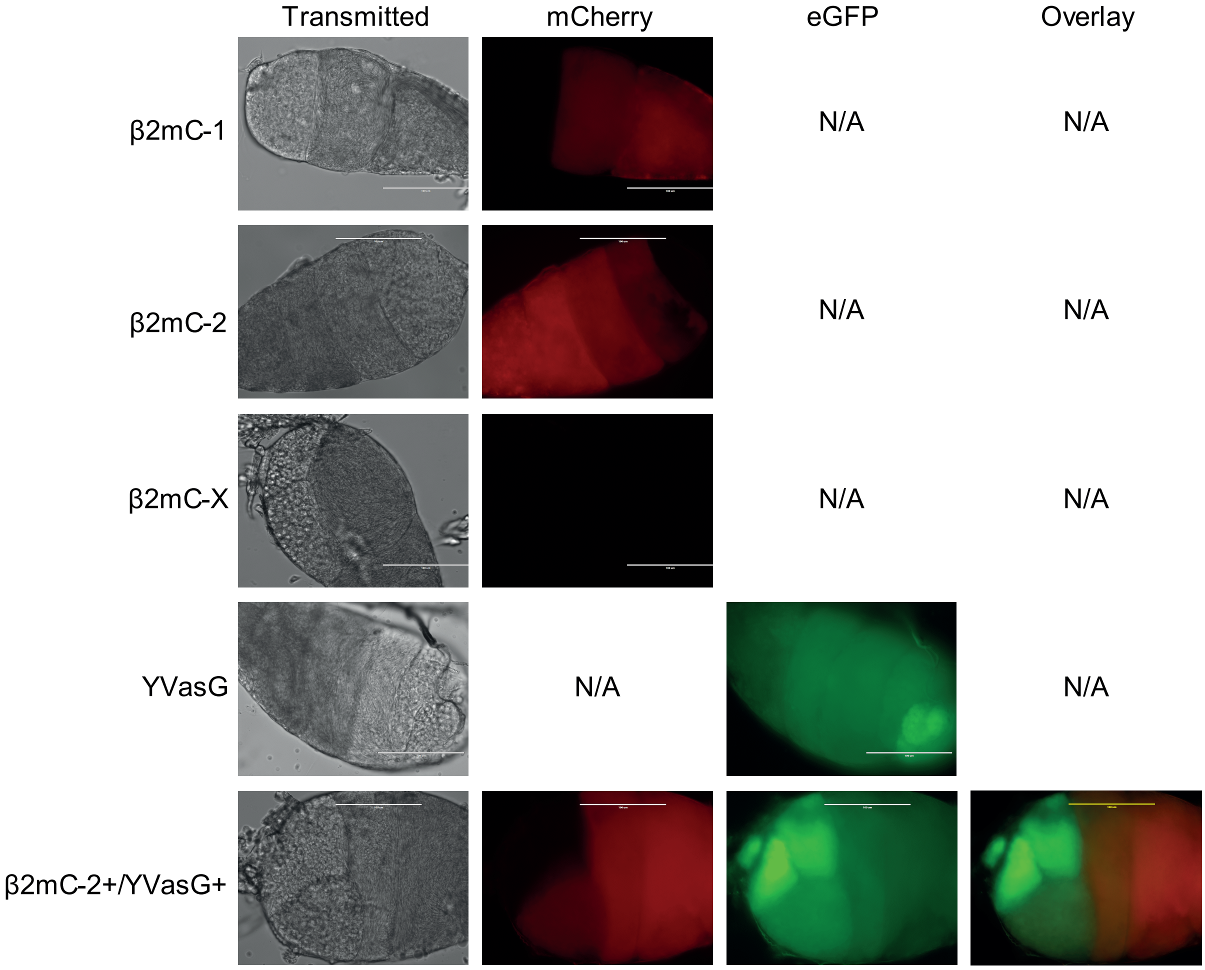

Images of testes dissected from 1-day old adult mosquitoes of each transgenic line. From left to right: transmitted, mCherry, eGFP channels and overlay of mCherry and eGFP fluorescent signal. Scale bars: 100 µm.

Supplementary Figure S2: Fluorescent activated cell sorting of β2mC-2⁺/YVasG⁺ testicular germline cell suspensions.

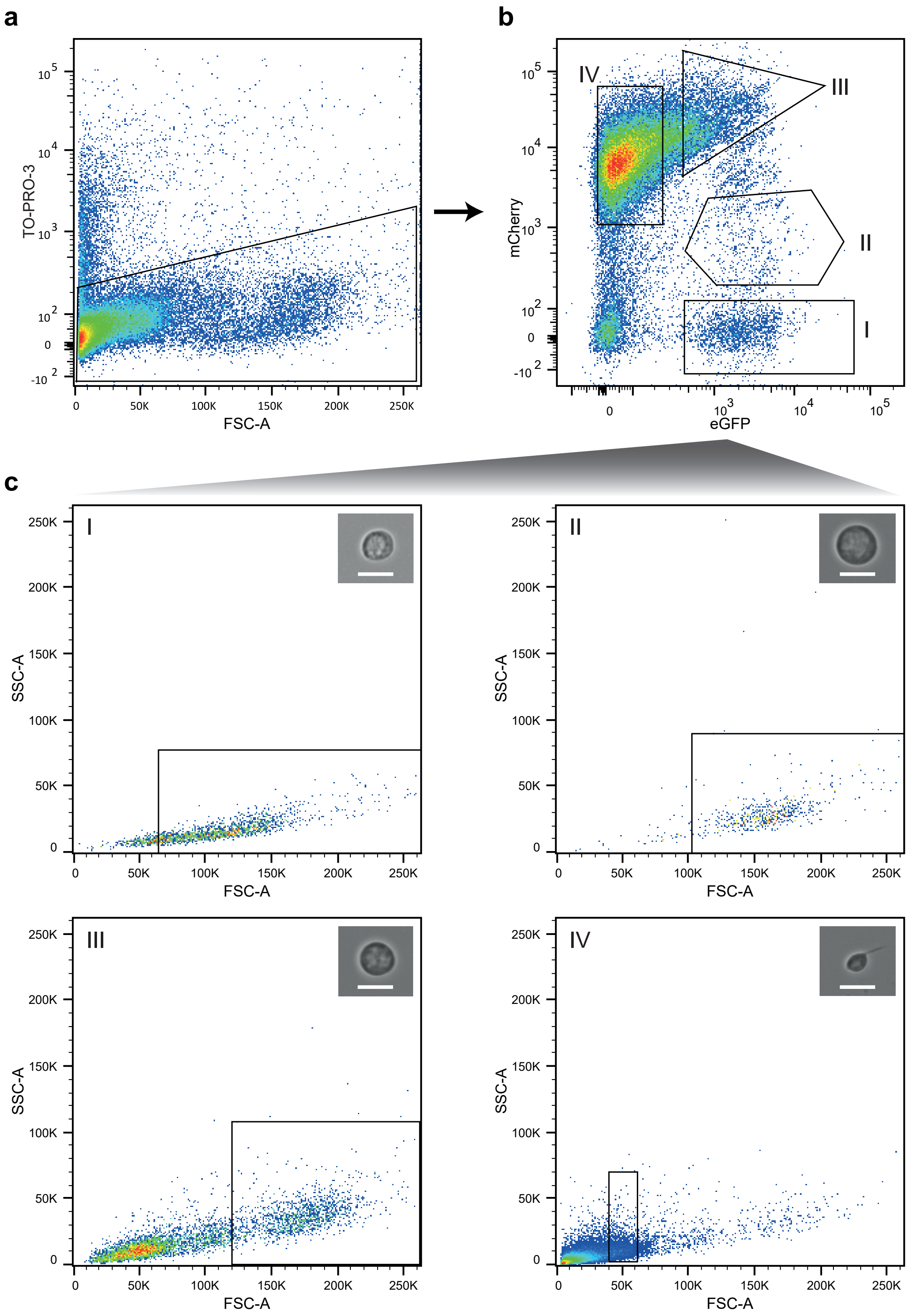

Flow cytometry dot plot of FlowJo biexponential scale values of **(a)** TO-PRO-3 iodide and FSC-A to

exclude dead cells and **(b)** mCherry and eGFP fluorescence intensity of live cells. **(c)** Linear FSC-A and SSC-A dot plots of the four selected populations indicating gates for cell sorting to exclude debris with low FSC-A. FSC-A/SSC-A gating was also applied to capture the majority of spermatocytes, excluding smaller spermatids, in population III; and to capture postmeiotic spermatids, excluding the smaller mature spermatozoa and the larger spermatocytes, in population IV. Indicative brightfield images of sorted cells are also shown for each sorted population. Scale bars: 10 µm.

Supplementary Figure S3: Expression profiles of β2-mCherry, vasa2:eGFP and endogenous *β2 tubulin* and *vasa* genes.

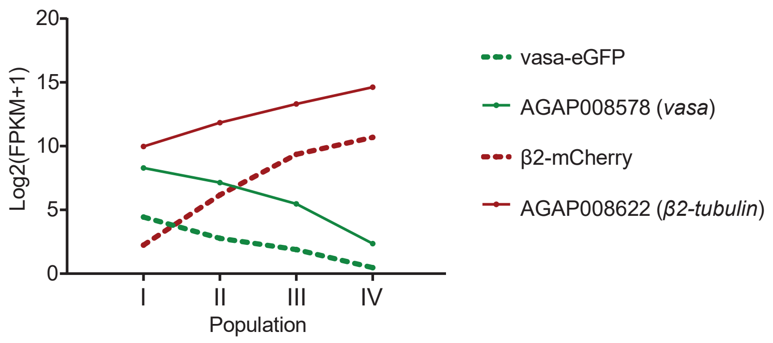

Line graph of vasa-eGFP, AGAP008578, β2-mCherry and AGAP008622 transcript levels across the four populations.

Supplementary Figure S4: Expression profiles of conserved meiotic genes and *D. melanogaster* orthologs.

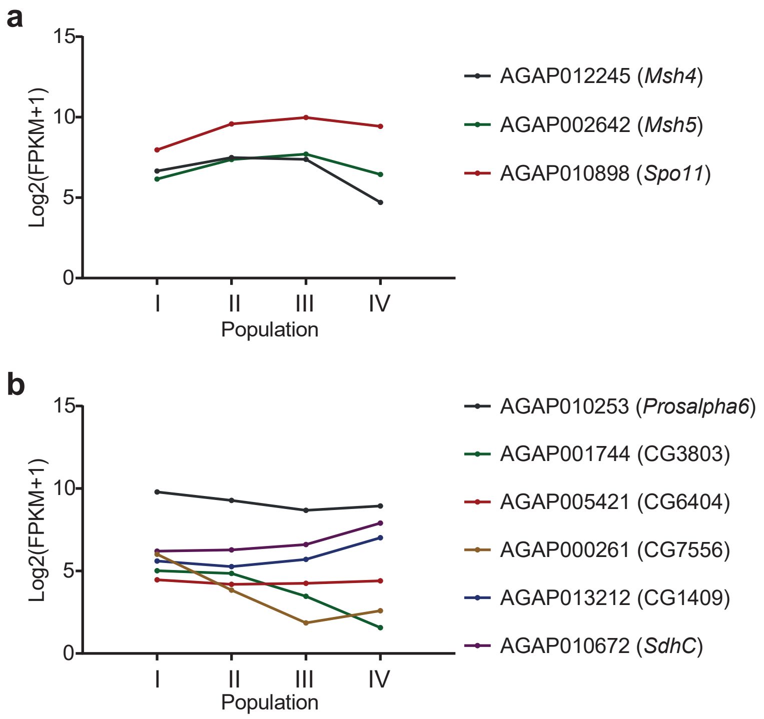

Expression levels of **(a)** conserved meiotic genes showing transcriptional peak in meiotic populations and **(b)** orthologues of *Drosophila melanogaster* testis genes previously tested through in situ hybridisation[24].

Supplementary Figure S5: Transcript isoform switching.

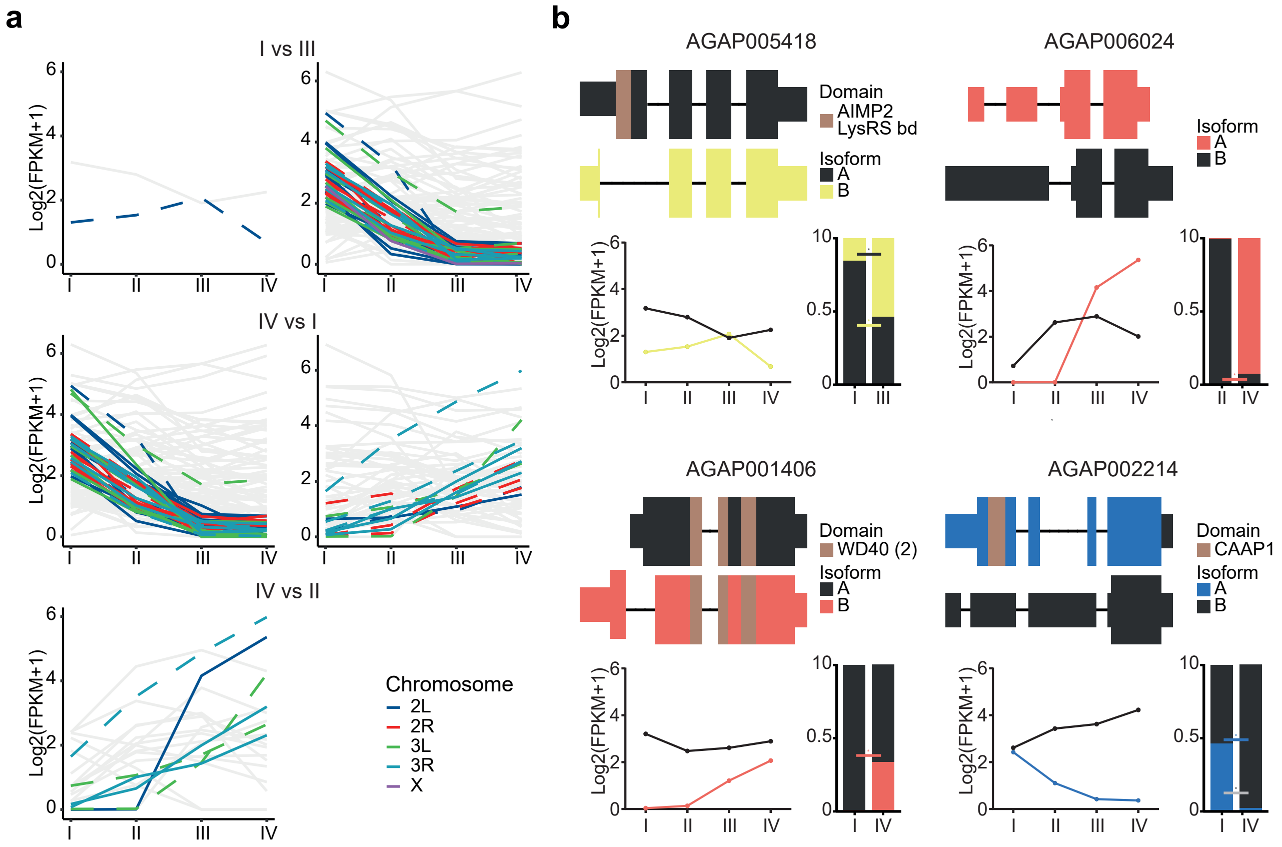

(a) Expression levels of significantly switched transcript isoforms differentially used among populations (adjusted *P* < 0.05). **(b)** Examples of population enriched (fold change higher than 2 and adjusted *P* < 0.05 after Sleuth analysis[41]) and differentially used transcript isoforms (adjusted *P*-value below 0.05 after IsoformSwitchAnalyzeR analysis[42]). Isoform A represents the annotated transcript and B the novel. The predicted functional features are shown at the top of each gene dataset where ticker rectangles indicate the coding sequence of the gene and domains with predicted function are indicated in light brown. Line graphs and bar charts represent respectively the expression profile and the usage across the four populations for each of the transcript isoform. Only genes that showed expression above 1 FPKM in at least one of the cell populations were included in the de novo transcriptome analysis.

Supplementary Figure S6: Germline expression dynamics of overrepresented biological processes in differentially expressed gene clusters.

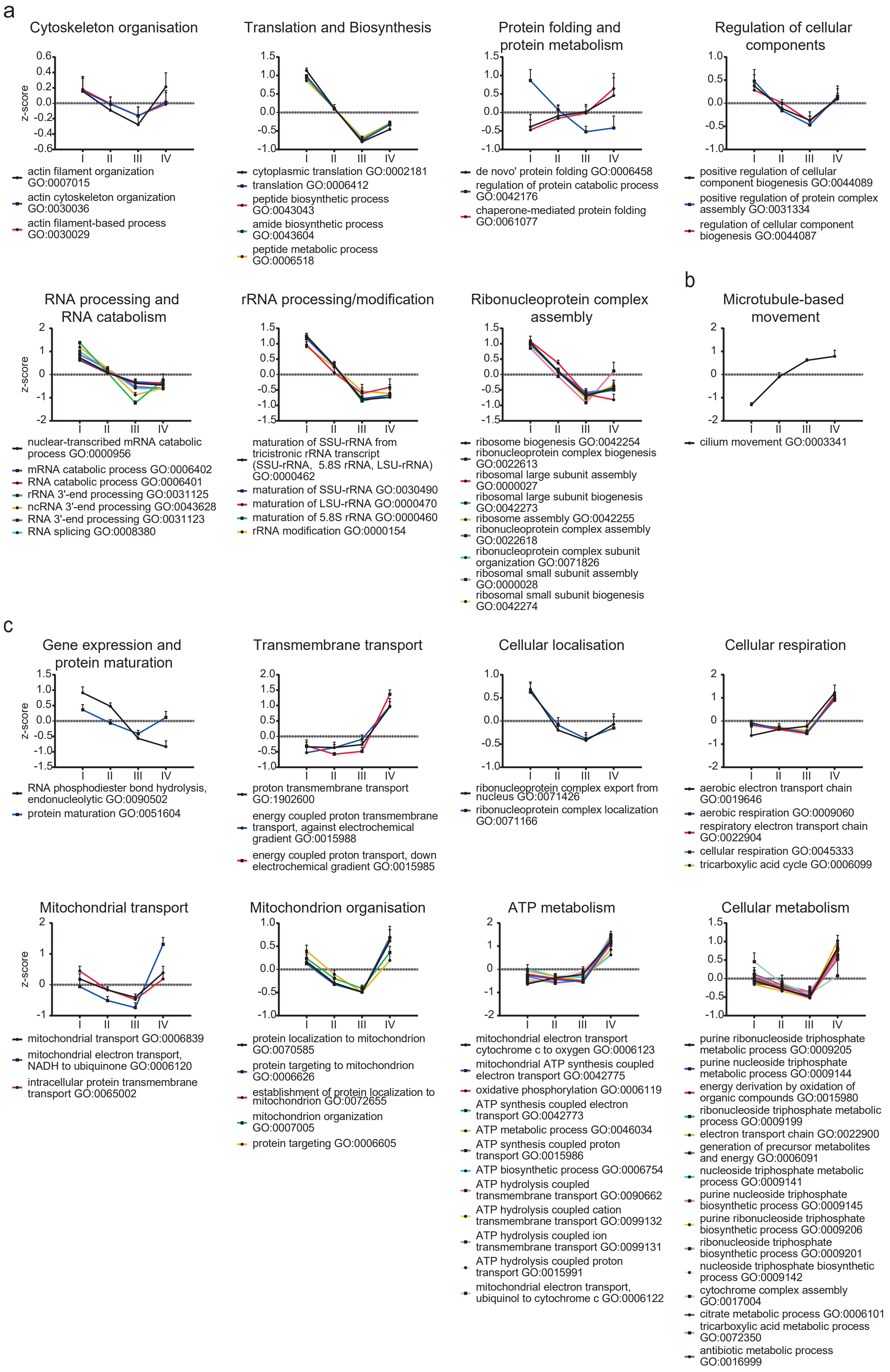

Average Z-score of genes associated to GO terms significantly enriched in each k-means cluster shown in **Fig. 3a**. **(a)** GO groups significantly enriched in the premeiotic clusters 1, 2 and 3. **(b)** GO term significantly enriched in the postmeiotic cluster 8. **(c)** GO groups significantly enriched in the postmeiotic clusters 9 and 10. Significance of the statistical overrepresentation test was analysed by applying Fisher's Exact test with False Discovery Rate correction.

Supplementary Figure S7: Germline expression dynamics of overrepresented biological processes in testes-enriched genes.

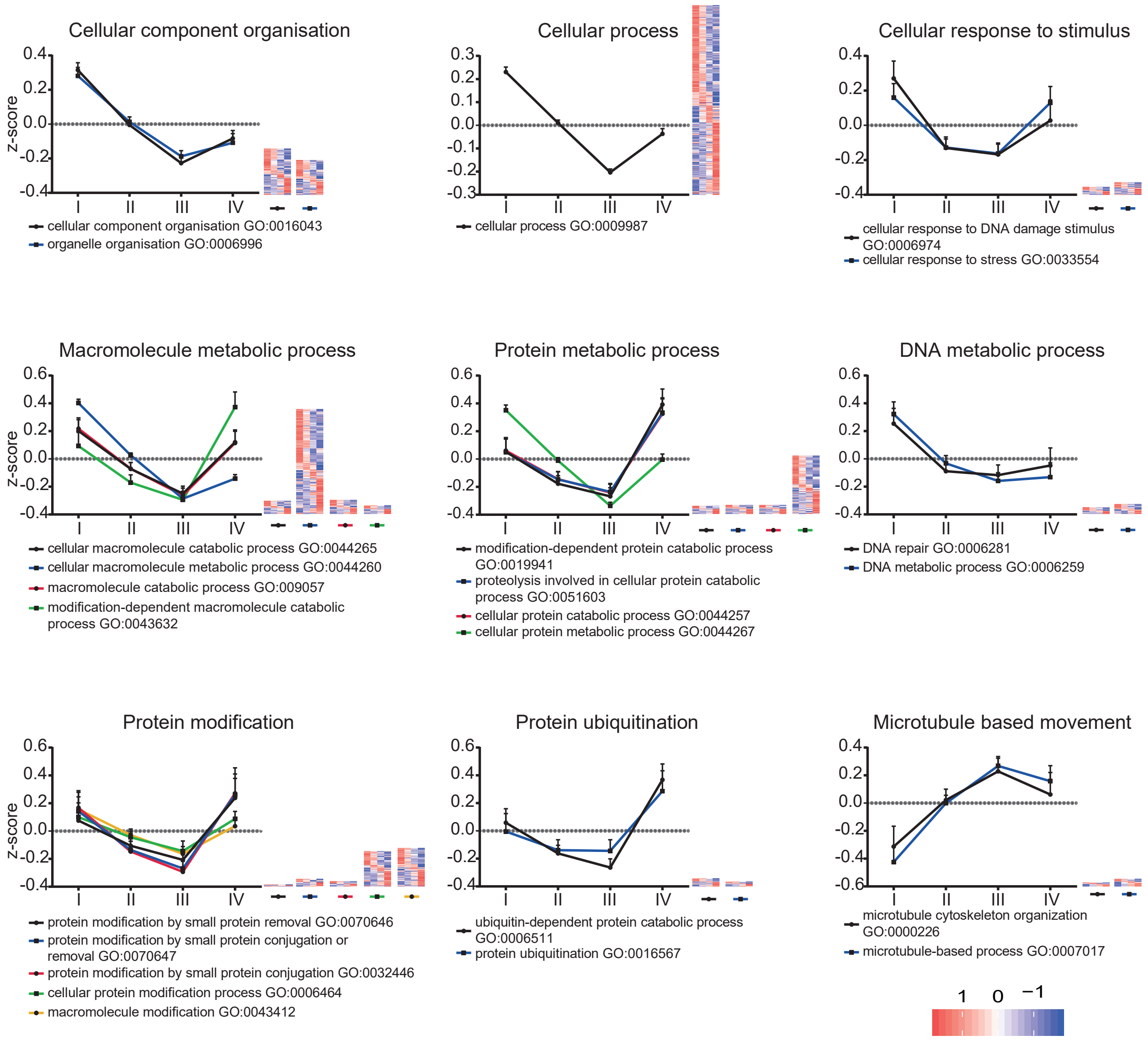

Average Z-score of genes associated to GO terms significantly overrepresented among testes-enriched genes based on tissue enrichment calculation (tau-value ≥ 0.8 and maximum expression in testis) and false-colour k-means clustering heatmap summarizing the expression patterns (as Z-score of log2(FPKM+1)) of genes included in each GO term.

Supplementary Figure S8: Germline expression dynamics of spermatogenesis-related KEGG metabolic pathways.

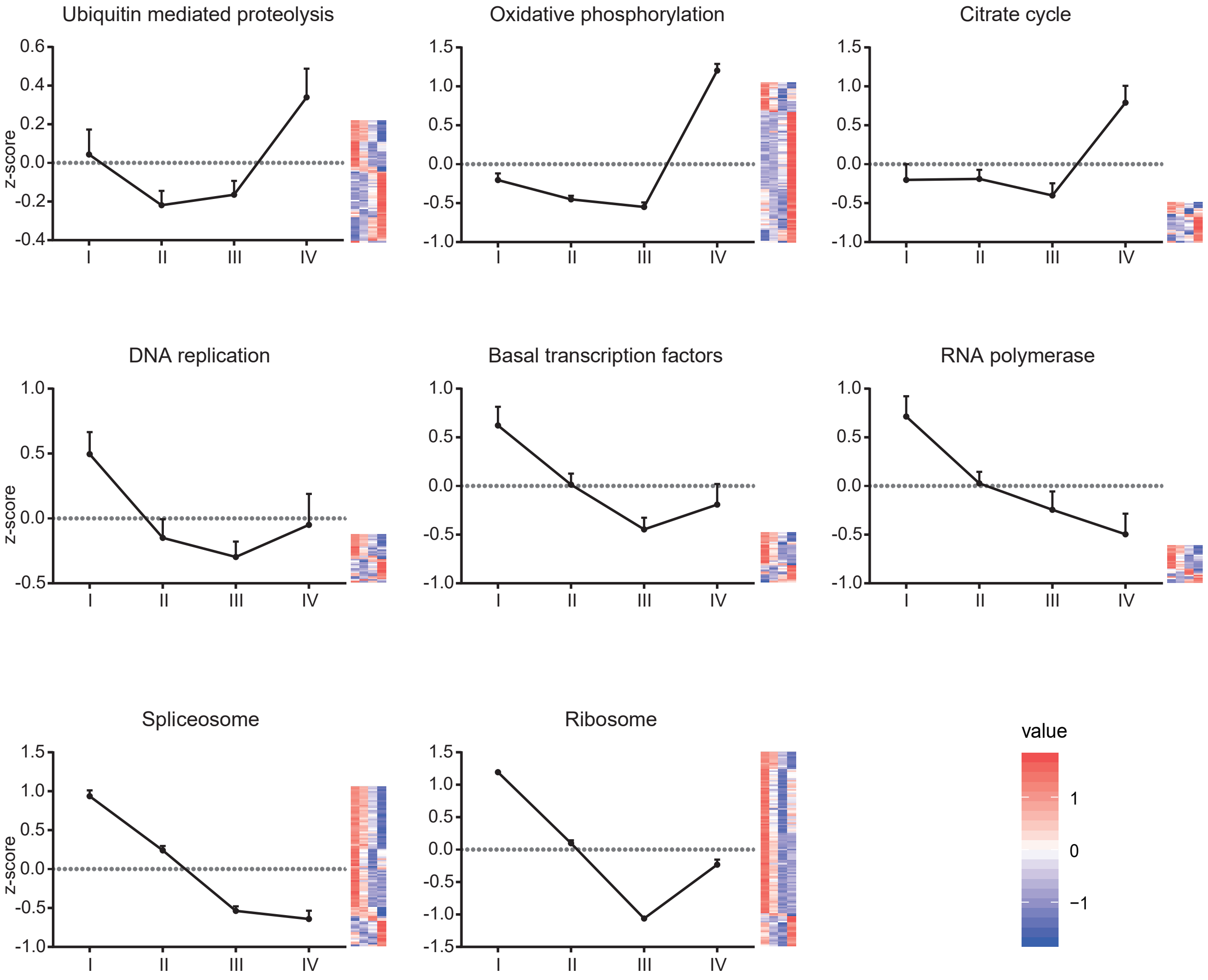

Average Z-score of genes associated to main KEGG metabolic pathways of relevance for male gametogenesis and false-colour k-means clustering heatmap summarizing the expression patterns (as Z-score of log2(FPKM+1)) of genes included in each KEGG pathway.
